# Ginsenoside Rh3 induces pyroptosis and ferroptosis through the Stat3/p53/NRF2 axis in colorectal cancer cells: Ginsenoside Rh3 has anti-colorectal cancer properties

**DOI:** 10.3724/abbs.2023068

**Published:** 2023-04-19

**Authors:** Yingchao Wu, Dajin Pi, Shuyao Zhou, Zhongjia Yi, Yangyang Dong, Wuhong Wang, Huan Ye, Yiliu Chen, Qian Zuo, Mingzi Ouyang

**Affiliations:** 1 School of Traditional Chinese Medicine Jinan University Guangzhou 510632 China; 2 College of Forestry and Landscape Architecture South China Agricultural University Guangzhou 510642 China; 3 Guangdong Metabolic Diseases Research Center of Integrated Chinese and Western Medicine Guangdong Pharmaceutical University Guangzhou 510006 China; 4 MOE Key Laboratory of Tumor Molecular Biology and Key Laboratory of Functional Protein Research of Guangdong Higher Education Institutes Institute of Life and Health Engineering College of Life Science and Technology Jinan University Guangzhou 510632 China

**Keywords:** pyroptosis, ferroptosis, ginsenoside Rh3, Stat3/p53/NRF2 axis, colorectal cancer

## Abstract

Ginsenoside Rh3 (GRh3) is a seminatural product obtained by chemical processing after isolation from Chinese herbal medicine that has strong antitumor activity against human tumors. However, its antitumor role remains to be elucidated. The aim of this study is to explore the mechanisms underlying the tumor suppressive activity of GRh3 from the perspective of pyroptosis and ferroptosis. GRh3 eliminates colorectal cancer (CRC) cells by activating gasdermin D (GSDMD)-dependent pyroptosis and suppressing solute carrier family 7 member 11 (SLC7A11), resulting in ferroptosis activation through the Stat3/p53/NRF2 axis. GRh3 suppresses nuclear factor erythroid 2-related factor 2 (NRF2) entry into the nucleus, leading to the decrease of heme oxygenase 1 (HO-1) expression, which in turn promotes NOD-like receptor thermal protein domain associated protein 3 (NLRP3) and caspase-1 expression. Finally, caspase-1 activates GSDMD-dependent pyroptosis. Furthermore, GRh3 prevents NRF2 from entering the nucleus, which suppresses SLC7A11, causing the depletion of glutathione (GSH) and accumulation of iron, lipid reactive oxygen species (ROS) and malondialdehyde (MDA), and eventually leading to ferroptosis in CRC cells. In addition, GRh3 effectively inhibits the proliferation of CRC cells *in vitro* and in nude mouse models. Collectively, GRh3 triggers pyroptotic cell death and ferroptotic cell death in CRC cells via the Stat3/p53/NRF2 axis with minimal harm to normal cells, showing great anticancer potential.

## Introduction

Colorectal cancer is a type of cancer with a high degree of malignancy; more than 1.9 million new colorectal cancer (including anus) cases and 935,000 deaths occurred in 2020, representing approximately one in 10 cancer cases and deaths [1]. The cornerstones of therapy for colorectal cancer (CRC) include surgery, neoadjuvant radiotherapy and auxiliary chemotherapy. Chemotherapy is still a common method of comprehensive treatment because of the difficulty in early diagnosis [ 2, 3] . Nevertheless, most patients have been reported to be refractory to chemotherapies, and antiapoptotic effects are among the causes of chemoresistance. Thus, it is critical to explore new strategies to better understand tumor biology and thereby improve clinical survival.

Programmed cell death includes apoptosis, necroptosis, ferroptosis, and pyroptosis [4]. Among the intrinsic forms of cell death, pyroptosis is a proinflammatory form of regulated cell death. Pyroptosis was originally described to occur in *Salmonella*-infected macrophages and is accompanied by Caspase-1 activation [ 5, 6] . Gasdermin family proteins are the executors of pyroptosis [7], which is characterized by cell swelling and rapid cell rupture, followed by the release of proinflammatory interleukin (IL)-1β, IL-18 and other cellular contents into the extracellular space. A recent study revealed that chemotherapy drugs can eliminate cancer cells through the induction of pyroptosis in cancer cells [7]. Pyroptosis has attracted growing attention because of its potential to increase the efficacy of cancer immunotherapy and eliminate cancer cells through the induction of pyroptosis [ 8, 9] . These results indicate that inducing pyroptosis in cancer cells may be an effective method for the treatment of tumors.

Ferroptosis is another type of regulatory cell death that differs from autophagy, apoptosis, necrosis and pyroptosis, and is caused by an overload of iron and reactive oxygen species (ROS) [10]. Recently, studies have shown the important role of ferroptosis in reversing tumor drug resistance, as it can inhibit phospholipid glutathione peroxidase 4 (GPX4) and the accumulation of lipid ROS in cells to trigger drug-resistant cancer cell death [ 11, 12] . Therefore, ferroptosis has great potential to become a new approach in antitumor therapy and compensate for some classic drugs, paving a new way for their application in the clinic [13]. It is of great significance to develop drugs that target ferroptosis in tumor cells.

Ginsenoside Rh3 (GRh3; C _36_H _60_O _7_, molecular weight: 604.8690), a seminatural triterpene with potent anticancer activity [14], is a bacterial metabolite of ginsenoside Rg5, which is the main component of hot-processed ginseng [15]. Ginseng has been used to treat inflammatory, autoimmune and malignant diseases for more than a hundred years [16]. Ginseng and its main ingredient ginsenoside possess potent antitumor activity in various human cancers [17]. Several natural products have been confirmed to inhibit tumor cell proliferation through pyroptosis and ferroptosis [ 18, 19] . The anticancer activity of GRh3 has been linked to the activation of caspase signaling [14]. However, the mechanisms underlying the tumor suppressive activity of GRh3 remain to be further explored.

In the present study, we demonstrated that GRh3 treatment robustly eliminated colorectal cancer cells through pyroptosis and ferroptosis. GRh3 activated gasdermin D (GSDMD)-dependent pyroptosis and suppressed xCT/SLC7A11, resulting in ferroptosis activation through the Stat3/p53/NRF2 axis. GRh3 suppressed NRF2 entry into the nucleus, leading to decreased HO-1 expression, which in turn promoted NLRP3 and caspase1 expression. Finally, caspase-1 activated GSDMD-dependent pyroptosis. Furthermore, GRh3 prevented NRF2 from entering the nucleus, which suppressed xCT/SLC7A11, causing the depletion of GSH and the accumulation of iron, lipid ROS and MDA, and eventually leading to ferroptosis in tumor cells.

## Materials and Methods

### GRh3 preparation

GRh3 (HPLC≥98%, Cat. #B21063) was purchased from Shanghai Yuanye Biotechnology Co., Ltd. (Shanghai, China) and dissolved in DMSO at a concentration of 50 mM for the *in vitro* experiments and phosphate-buffered saline (PBS) at a concentration of 50 mg/mL for the *in vivo* experiments. The GRh3 solution was stored at ‒80°C for subsequent use. In the *in vitro* experiments, GRh3 was dissolved in DMSO and medium, and the final concentration of DMSO in medium was ≤0.32% (v/v).

### Other chemicals and reagents

Dulbecco’s modified Eagle’s medium (DMEM; Cat. #11965092) with high glucose, L-glutamine, and phenol red (Cat. #25200072), fetal bovine serum (FBS; Cat. #10270106), penicillin/streptomycin (Cat. #10378016), PBS (Cat. #10010023), trypsin-EDTA, and 3-(4,5-dimethylthiazol-2-y1)-2,5-diphenyltetrazolium bromide (MTT; Cat. #V13154) were supplied by Gibco (Carlsbad, USA). Giemsa staining solution (Cat. #C0133) was purchased from Beyotime Institute of Biotechnology (Shanghai, China). Belnacasan (VX-765; Cat. #5313720001), ferrostatin-1 (Fer-1; Cat. #SML0583), Z-VAD-FMK (Z-VAD; Cat. #V116), 3-methyladenine (3-MA; Cat. #M9281), necrostatin-1 (Nce-1; Cat. #N9037), and pifithrin-α (PFT-α; Cat. #P4359) were purchased from Sigma-Aldrich (Darmstadt, Germany). Primary antibodies against Stat3 (Cat. #9139T), phospho-Stat3 (P-Stat3; Cat. #9145T), NRF2 (Cat. #12721T), histone H3 (Cat. #4499T), Ki-67 (Cat. #9449T), CD206 (Cat. #24595T), and GAPDH (Cat. #5174S), as well as HRP-conjugated anti-rabbit IgG (Cat. #7074P2) and anti-mouse IgG (Cat. #7076P2) secondary antibodies were purchased from Cell Signaling Technology (Danvers, USA). Primary antibodies against p53 (Cat. #60283-2-Ig), HO-1/HMOX1 (HO-1; Cat. #10701-1-AP), Caspase-1 (Cat. #22915-1-AP), gasdermin D (GSDMD; Cat. #20770-1-AP), xCT/SLC7A11 (SLC7A11; Cat. #26864-1-AP), and GPX4 (Cat. #67763-1-Ig) were purchased from Proteintech (Wuhan, China). The primary antibody against NLRP3 (Cat. #EPR23094-1) was purchased from Abcam PLC (Cambridge, UK).

### Cell culture

The human CRC cell lines HT29, HCT116, SW620, DLD1 and RKO, and the human normal colorectal cell line HCoEpiC were obtained from the Life Science and Technology College, Jinan University (Guangzhou, China). All cells were identified by short tandem repeat (STR). The cells were cultured in DMEM supplemented with 10% FBS, 100 U/mL penicillin and 100 μg/mL streptomycin in a 5% CO _2_ incubator (Thermo Fisher Scientific, Waltham, USA) at 37°C. The medium was changed every 72 h, and the cells were routinely subcultured when they reached 90% confluence. Logarithmic growth phase CRC cells and human normal colorectal cells were used in the experiments. If the experimental process required treatment with inhibitors, the working concentration of VX-765 was 20 μM, the working concentration of Fer-1 was 2 μM, the working concentration of Z-VAD was 60 μM, the working concentration of 3-MA was 2 nM, the working concentration of Nce-1 was 40 μM, and the working concentration of PFT-α was 20 μM, and the cells were pretreated with these inhibitors for 4 h. The cells were used in the subsequent experiments 48 h after the treatment with or without GRh3 unless stated otherwise.

### Analysis of cell viability

Cell viability was measured by MTT colorimetric assay. CRC cells or HCoEpiC cells were seeded in 96-well plates (8×10 ^3^ or 5×10 ^3^ cells/well in 100 μL) and cultured for 12 h. To determine different dose-dependent effects, the original medium was discarded, and 100 μL of DMEM-diluted GRh3 solution was added to each well at final concentrations of 0, 10, 20, 40, 80, and 160 μM or the cells were pretreated with different inhibitors before the addition of GRh3-containing medium. The cells were cultured with GRh3-containing medium for 24 or 48 h before the MTT colorimetric assay was performed. After culture, 20 μL of MTT solution (5 mg/mL; Cat. #V13154; Gibco) was added to each well. After the cells were cultured for 4 h at 37°C, the supernatant was discarded, 150 μL of DMSO was added to each well, and the sample was stirred well for 15 min. The absorbance of each well was then measured with a microplate reader (BioTek Epoch, Vermont, USA) at a wavelength of 490 nm. The recorded optical density (OD) values represent the cell vitality. Then, the cell viability rate was calculated using the following equation: cell viability rate (%)=(OD _Sample_/ OD _Control_) ×100%. These experiments were performed in triplicate.

### Colony formation assay

HT29 and HCT116 cells were seeded in 6-well plates (500 cells/well in 2000 μL) and cultured for approximately 14 days. Colonies with more than 50 cells per colony were treated with 0.16% (v/v) DMSO or GRh3-containing medium (20, 40, and 80 μM) for 48 h. Subsequently, the medium was removed, and the cells were washed twice with PBS. After the cells were fixed with 4% paraformaldehyde, the colonies were stained with Giemsa stain for 5 min, and images were taken to record the results. These experiments were performed in triplicate, and ImageJ software was used for quantitative analysis.

### Cell pyroptosis assay

Pyroptosis was determined by using an Annexin V-FITC Apoptosis Detection kit (Cat. #C1062L; Beyotime Institute of Biotechnology) according to the manufacturer’s protocol. Briefly, a single-cell suspension was incubated with 5 μL annexin V-FITC and 10 μL propidium iodide (PI) for 15 min at room temperature (20‒25°C). The cells were analyzed by flow cytometry. In total, 10,000 events were recorded for each analysis using the NovoExpress software (Agilent Technologies, Inc., Santa Clara, USA).

### Assessment of cell growth morphology

HT29 and HCT116 cells were seeded in 6-well plates (50,000 cells/well in 2000 μL) and cultured for 12 h. After 12 h of culture, the original medium was discarded, and 2 mL of DMEM-diluted GRh3 solution was added to each well at a final concentration of 0, 20, or 40 μM. Two wells containing 40 μM GRh3 were pretreated with VX-765 or Fer-1. After 48 h of culture, the medium was discarded, fresh complete medium was added, and images were taken immediately under an inverted microscope at 400× magnification (Nikon, Tokyo, Japan).

### Enzyme-linked immunosorbent assay (ELISA)

Cells were treated with GRh3 for the indicated time. The culture media were collected and centrifuged to remove any cell debris or suspended cells. Serum was collected from sacrificed mice. IL-1β and IL-18 released into the culture medium or in the serum were measured by using a human IL-1β ELISA kit (Cat. #70-EK101B-96; Multisciences Biotech, Hangzhou, China) and human IL-18 ELISA kit (Cat. #70-EK118-96; Multisciences Biotech), respectively.

### Human CRC xenograft mouse model

Forty BALB/c nude mice (five-week old, 15± 1 g) were purchased from the Model Animal Research Center of Nanjing University (Nanjing, China). All animal experiments (Approval No. IACUC-20201231-04) were conducted according to the relevant laws and institutional guidelines with the approval of the Animal Ethics Committee of Jinan University [SYXK (YUE) 2017-0174]. After acclimation for 10 days, the mice were randomly divided into two groups and inoculated (sc) with HT29 or HCT116 cells (1×10 ^7^ cells/ 100 μL each) in the right forelimb pit. When the tumor grew to 50 mm ^3^, the mice in each group were randomly assigned into the following two subgroups ( *n*=10): the control group (solvent) and the GRh3-treated group (ig, 20 mg/kg/d). The body weights and tumor volumes were measured every 7 days, and the tumor size was calculated using the following equation: tumor volume=length×width ^2^×1/2. Twenty-one days after the treatment, the mice were anaesthetized with pentobarbital (ip, 150 mg/kg) and sacrificed by cervical dislocation. The serum was retained, and the xenograft tumor tissues, liver and kidney were resected for subsequent experiments.

### Hematoxylin and eosin (HE) staining

Fresh liver, kidney, and xenograft tumor tissues were fixed with 4% paraformaldehyde, dehydrated, embedded in paraffin, cut into 5 μm slices, stained with hematoxylin for 30 min and eosin for 5 min, vitrified with xylene, and sealed with neutral resin. The stained slices were observed and photographed under a light microscope (Nikon) at 200× magnification.

### Western blot analysis

The cell and xenograft tumor tissue samples were lysed in RIPA buffer (Cat. #P0013B; Beyotime Institute of Biotechnology) containing 1 mM phenylmethylsulfonyl fluoride (PMSF; Cat. #ST505; Beyotime Institute of Biotechnology) and 1× protease and phosphatase inhibitor cocktail (Cat. #P1045; Beyotime Institute of Biotechnology) on ice. If nuclear protein isolation was needed, it was performed using a Nuclear and Cytoplasmic Protein Extraction kit (Cat. #P0027; Beyotime Institute of Biotechnology). The lysates were then centrifuged at 12,000 *g* for 15 min at 4°C, and the protein concentration in the supernatant was determined using an Enhanced BCA Protein Assay Kit (Beyotime Institute of Biotechnology). Equal amounts of protein were separated via SDS-PAGE and transferred to polyvinylidene fluoride (PVDF) membranes (Cat. #IEVH00005; Merck KGaA, Darmstadt, Germany). Next, the PVDF membranes were blocked with Tris-buffered saline plus Tween-20 (TBST) containing 5% skim milk for 1 h and incubated with the indicated primary antibodies, including mouse anti-Stat3 (1:1000) and anti-p53 (1:2000) and rabbit anti-P-Stat3 (1:1000), anti-NRF2 (1:1000), anti-Histone H3 (1:1000), anti-HO-1 (1:1000), anti-NLRP3 (1:1000), anti-Caspase-1 (1:1000), anti-GSDMD (1:1000), anti-xCT/SLC7A11 (1:1000), anti-GPX4 (1:1000), and anti-GAPDH (1:3000). Then, the membranes were incubated with the corresponding secondary antibodies (1:5000) for 60 minutes at room temperature. PVDF membranes were washed three times with TBST solution, and visualized by Beyotime′s hypersensitive ECL kit (Cat. #P0018S; Beyotime Institute of Biotechnology). Finally, density values of protein bands were captured and documented through a gel image analysis system (ChemiDoxTM; Bio-Rad, Hercules, USA). GAPDH was used as the loading control.

### Measurement of intracellular and xenograft tumor tissue GSH concentrations

Fresh xenograft tumor tissues or cells that were incubated in medium containing different concentrations of GRh3 for 48 h were collected, and the intracellular and tissue GSH concentrations were measured using the GSH and GSSG assay kits (Cat. #S0053; Beyotime Institute of Biotechnology) according to the manufacturer’s protocols. The experimental data were determined with a microplate reader. These experiments were performed in triplicate and the results were expressed as a percentage relative to the blank/control group.

### Measurement of intracellular and xenograft tumor tissue iron concentrations

The iron concentrations in the cells or tissues were measured using an iron assay kit (Cat. #MAK025; Sigma-Aldrich) according to the manufacturer’s instructions. Briefly, cells (2×10 ^6^ cells) were treated with different concentrations of GRh3 in medium for 48 h, and fresh xenograft tumor tissue (10 mg) was rapidly homogenized in 5 volumes of iron assay buffer. The samples were centrifuged at 16,000 *g* for 10 min at 4°C to remove insoluble material. A microplate reader was used to measure the absorbance at 593 nm. These experiments were performed in triplicate, and the results were expressed in nM.

### Measurement of intracellular lipid ROS concentrations

C11-BODIPY (Cat. #D3861; Thermo Fisher Scientific) was used to measure the intracellular lipid ROS concentrations according to the manufacturer’s instructions. Briefly, after being treated with different concentrations of GRh3 in medium for 48 h, the cells were cultured with 100 μMC11-BODIPY for 30 min at 37°C. The samples were washed twice with PBS. Then, the fluorescence intensities were measured at an emission wavelength of 510 nm and an excitation wavelength of 488 nm using a fluorescence microplate reader. These experiments were performed in triplicate and the results were expressed as a percentage of the fluorescence intensity relative to that of the blank group.

### Measurement of xenograft tumor tissue ROS concentrations

A tissue ROS assay kit (Cat. #BB-470532; Bestbio, Shanghai, China) was used to measure the xenograft tumor tissue ROS concentrations. Briefly, after fresh xenograft tumor tissues were collected, the ROS concentration in the xenograft tumor tissues was measured with a fluorescence microplate reader (BioTek) according to the kit instructions. These experiments were performed in triplicate and the results were expressed as a percentage of the fluorescence intensity relative to that of the control group.

### Measurement of intracellular and xenograft tumor tissue MDA concentrations

A Lipid Peroxidation MDA Assay Kit (Cat. #S0131S; Beyotime Institute of Biotechnology) was used to measure the MDA concentrations in cells or tissues. In brief, cells were treated with different concentrations of GRh3 in medium for 48 h and fresh xenograft tumor tissues were collected. According to the kit’s protocol, the experimental data were analysed with a microplate reader. These experiments were performed in triplicate and the results were expressed in μmol/mg.

### RNA-Seq

Total tumor tissue RNA was extracted by using TRIzol reagent (Life Technologies, Grand Island, USA). After digestion with RNase-free DNase I (Takara, Dalian, China), complementary DNA was prepared by using a RevertAid First Strand cDNA Synthesis kit (Thermo Fisher Scientific). Sequencing was performed by using an Illumina HiSeq platform (San Diego, USA). Differentially expressed genes with a fold change ≥ 2 were determined by the NOISeq method. Differential (cluster and deglist) and enrichment (GO and KEGG) analyses were completed by the analysis platform of Beijing Novogene Technology (Beijing, China). The original datasets of RNA-seq can be obtained by accessing the NCBI Sequence Read Archive (URL: https://www.ncbi.nlm.nih.gov/sra/PRJNA862091).

### Terminal-deoxynucleoitidyl transferase mediated nick end labeling (TUNEL) staining

Formalin-fixed xenograft tumor tissue samples were embedded in paraffin, and the paraffin-embedded samples were then cut into serial sections (4 μm thick). After dewaxing, the sections were stained using TUNEL apoptosis detection kit (Cat. #ATK00001; AtaGenix, Wuhan, China) according to the instruction from this manufacturer. Then, the samples were stained with 4′,6-diamidino-2-phenylindole (DAPI; 0.05 μg/mL; Cat. #G1012; Servicebio, Wuhan, China) for 10 min and sealed after rinsing three times with PBS. Fluorescent images were captured at 400× magnification under a fluorescence microscope (NIKON Eclipse ci, Tokyo, Japan).

### Immunohistochemical (IHC) staining

Tissue sections were performed according to the above method. These sections were immunostained with the indicated primary antibodies, including anti-Ki-67 (1:800), anti-p53 (1:1600), anti-Caspase-1 (1:100), anti-GSDMD (1:100), anti-SLC7A11 (1:50), anti-GPX4 (1:500), and anti-CD206 (1:200), followed by incubation with the corresponding secondary antibodies (1:3000). Images were captured under a light microscope (Leica, Wetzlar, Germany).

### Statistical analysis

Statistical analysis was performed using Student-Newman-Keuls (S-N-K) and ANOVA with SPSS version 13.0 software (SPSS Inc., Chicago, USA) or GraphPad Prism 9 (GraphPad Software, San Diego, USA). Data are presented as the mean±standard deviation (SD). A *P* value<0.05 was considered statistically significant.

## Results

### GRh3 inhibits CRC cell proliferation *in vitro* and *in vivo*

**Figure 1:**
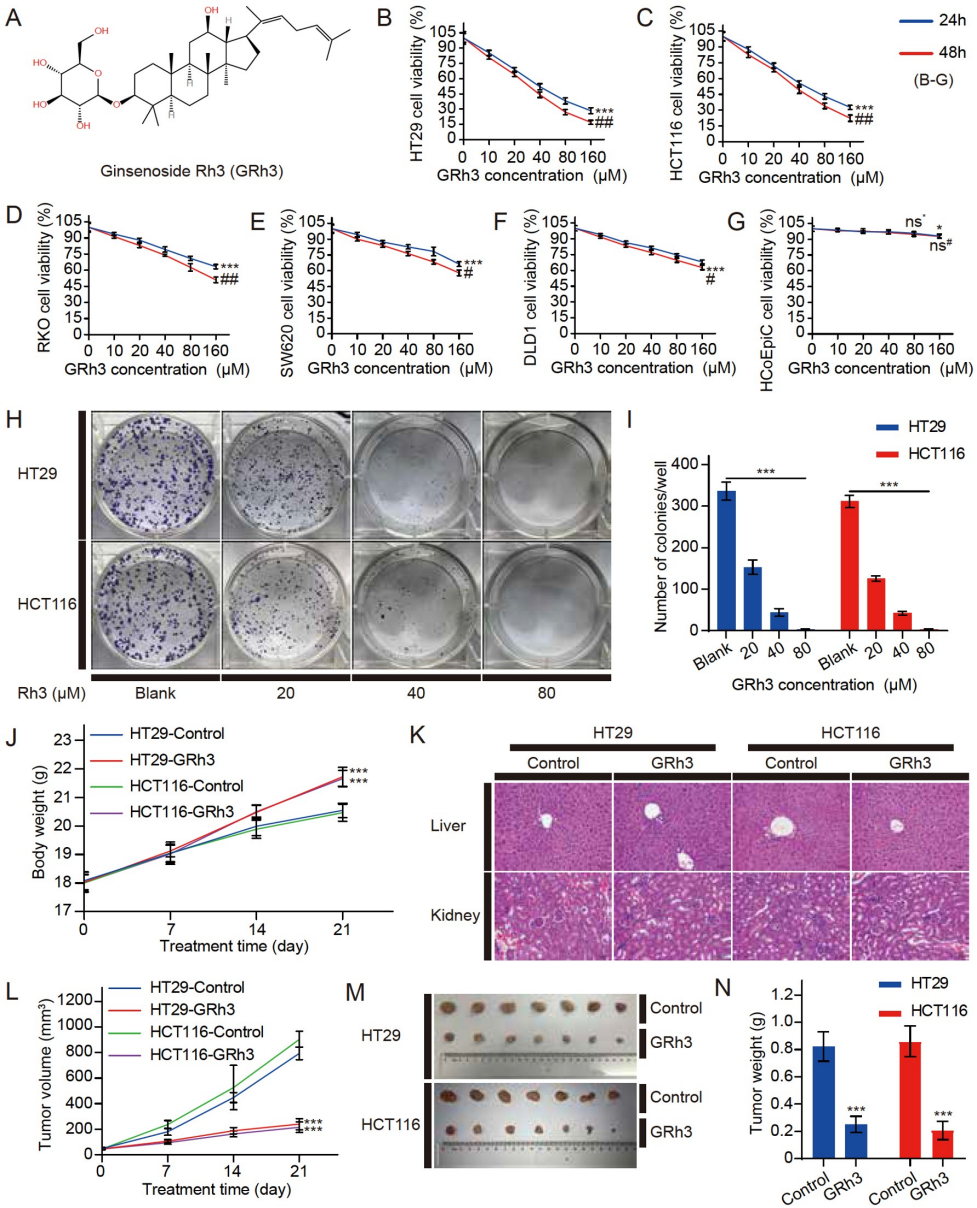
Figure 1 GRh3 significantly inhibits the proliferation of CRC cells *in vivo* and *in vitro* (A) The chemical structure of GRh3. (B‒G) The cell viability of CRC cells (HT29, HCT116, RKO, SW620, and DLD1 cells in that order) and HCoEpiC cells treated with different concentrations of GRh3 in medium for 24 and 48 h ( n=3). (H,I) Giemsa-stained colonies were observed under an inverted microscope and quantified ( n=3). HT29 and HCT116 human CRC xenograft mouse models were treated with solvent or GRh3 (20 mg/kg/d). (J) Body weight was measured every 7 days ( n=10). (K) HE staining of liver and kidney tissues. Scale bar= 100 μm. (L) Tumor size was measured every 7 days ( n=10). (M) Representative images of HT29 and HCT116 xenograft tumor tissues from the control (solvent) and GRh3-treated groups. (N) Xenograft tumor tissue weight after 21 days of treatment ( n=10). Data are presented as the mean±SD of triplicate experiments. (B‒G) * P<0.05, *** P<0.001 compared with the blank group (0 μM GRh3); ## P<0.01, ### P<0.001 compared with the 24 h control group; (I,J,L,N) *** P<0.001 compared with the blank/control group.

The chemical structure of GRh3 is shown in Figure 1A. To determine whether GRh3 can inhibit CRC cell proliferation, we first established an *in vitro* model of CRC using HT29, HCT116, RKO, SW620, and DLD1 cells. The MTT colorimetric assay revealed that GRh3 could significantly inhibit CRC cell proliferation. GRh3 dose-dependently and time-dependently inhibited the proliferation of four types of colon cancer cells compared with that in the blank or control group (0 μM GRh3, 0.32% DMSO or 160 μM GRh3, 24 h) ( Figure 1B‒F). Moreover, at concentrations below 80 μM, GRh3 had a limited effect on human normal colorectal cells, and increasing the treatment time did not significantly inhibit normal cell viability ( Figure 1G). The results showed that HT29 and HCT16 cells were more sensitive to GRh3; thus, we selected these two cell lines for the subsequent experiments. In addition, the number of clones of HT29 and HCT116 cells was significantly decreased by GRh3 in a dose-dependent manner ( Figure 1H,I).

Then, to determine whether GRh3 can inhibit growth *in vivo*, we inoculated HT29 or HCT116 cells into nude mice to establish a xenograft mouse model of CRC. The mice were injected with solvent or GRh3 (20 mg/kg/d). After 21 days of treatment, the xenograft tumor size in the GRh3 group was significantly reduced compared with that in the control group ( Figure 1L‒N). However, there was a significant difference in body weight between the control and GRh3 groups ( Figure 1J). The body weight in the GRh3 treatment group was significantly higher than that in the solvent treatment group. Meanwhile, HE staining showed that GRh3 caused no significant damage to the liver and kidney, such as a disordered architecture of the hepatic lobule and enlargement of the glomerulus ( Figure 1K), suggesting that GRh3 has fewer toxic side effects. Therefore, these data suggest that GRh3 can significantly suppress CRC *in vivo* and *in vitro* without damaging organisms.

### Inhibition of CRC cell proliferation by GRh3 is associated with pyroptosis and ferroptosis and is closely related to the intracellular signal transduction/p53/NRF2 axis

**Figure 2:**
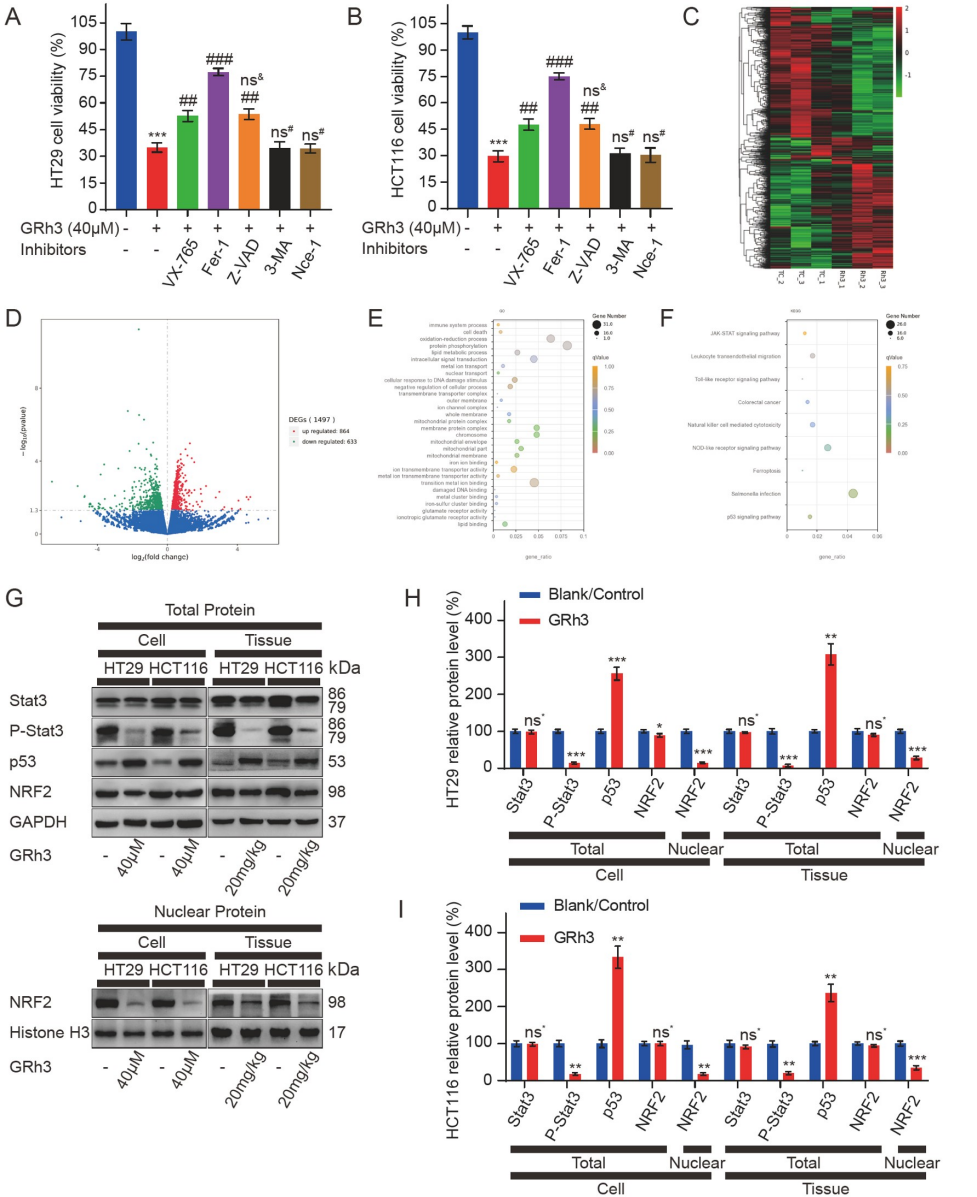
Figure 2 GRh3 induces pyroptosis and ferroptosis in CRC cells and affects the Stat3/p53/NRF2 axis (A,B) The viabilities of HT29 and HCT116 cells. HT29 and HCT116 cells were pretreated with VX-765 (pyroptosis inhibitor), Fer-1 (ferroptosis inhibitor), Z-VAD (apoptosis inhibitor), 3-MA (autophagy inhibitor), or Nce-1 (necrosis inhibitor) for 4 h and then incubated with 40 μM GRh3 for 48 h. (C) Heatmap showing the cluster of differentially expressed genes in the GRh3 treatment and tumor control groups. Up- and down-regulated genes are represented in red and green, respectively, n=3. (D) Volcano plot of differential expression signals in the GRh3 treatment and tumor control groups, n=3. (E) Bubble map of the GO enrichment analyses of differentially expressed genes in HT29 xenograft tumor tissue. The GeneRatio represents the degree of enrichment. The node size shows the number of selected genes, and the color scale represents padj, n=3. (F) Bubble map of KEGG pathway enrichment analyses of differentially expressed genes in HT29 xenograft tumor tissue. The GeneRatio represents the degree of enrichment. The node size shows the number of selected genes, and the color scale represents padj, n=3. (G) Representative western blots of Stat3/p53/NRF2 axis-related protein expression in HT29 and HCT116 cells treated with GRh3 in vitro or in vivo. (H,I) Relative expression levels of Stat3/p53/NRF2 axis-related proteins in HT29 and HCT116 cells. Data are presented as the mean±SD. * P<0.05, ** P<0.01, *** P<0.001, compared with the blank/control group (0 μM or 0 mg/kg GRh3); ## P<0.01, ### P<0.001, compared with the 40 μM GRh3 group.

To elucidate the functional role of GRh3 in inhibiting CRC, we used different pathway inhibitors to explore the effect of GRh3 on cell death. GRh3 significantly inhibited the growth of HT29 and HCT116 cells, and this effect was not reversed by inhibitors of autophagy or necrosis. Moreover, the inhibitory effects on cell proliferation were reversed by pyroptosis, ferroptosis, and apoptosis inhibitors ( Figure 2A,B). To explore the mechanism underlying GRh3’s antitumor effect *in vivo*, RNA-seq was performed using HT29 xenograft tumor tissue (Due to the financial problems and practical application scenarios of drugs, only HT29 xenograft tumor tissue samples were analyzed in this study). According to the transcriptome sequencing results, we conducted a differential analysis and found that approximately 5.65% (1497/26473) of gene expression was changed (up or downregulated) after GRh3 treatment compared with that after the control treatment ( Figure 2C,D). The results were analyzed by GO and KEGG pathway enrichment analyses. We found that the expression of the p53 signaling pathway and targets related to inflammation and ferroptosis in HT29 xenograft tissues changed after GRh3 treatment. In addition, we noted changes in biological processes, such as oxidative stress and nuclear translocation. ( Figure 2E,F). To verify the effects of GRh3 treatment on the above related targets and pathways, we analyzed the protein expression levels in cultured cells and xenograft tumor tissues *in vitro*. Western blot analysis showed that GRh3 treatment decreased the expression level of p-Stat3 and increased the expression level of p53 in the total protein, but there was no significant difference in the expression levels of Stat3 and NRF2. We further isolated the nucleus and found that the expression level of NRF2 in the nucleus was significantly reduced by GRh3 treatment ( Figure 2G‒I). Therefore, these data suggest that GRh3 inhibits CRC cell proliferation through pyroptosis or apoptosis and ferroptosis, which may be related to the role of the Stat3/p53/NRF2 axis.

### GRh3 induces pyroptosis in CRC cells *in vitro*

**Figure 3:**
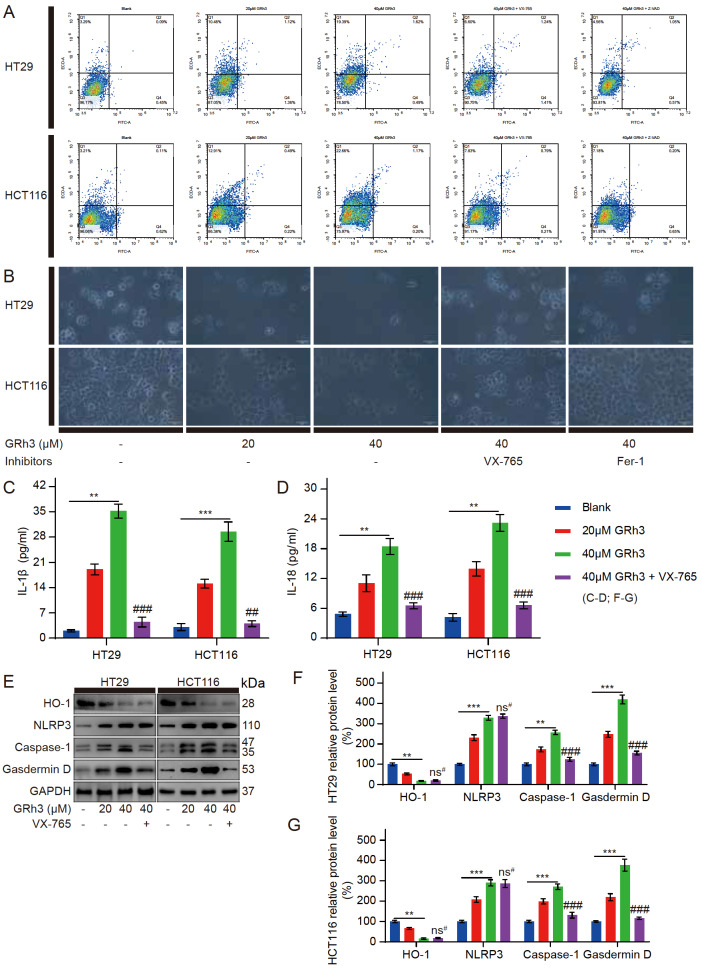
Figure 3 GRh3 induces pyroptosis in HT29 and HCT116 cells (A) Frequencies of pyroptotic cells treated with different concentrations of GRh3 in medium with or without VX-765 (pyroptosis inhibitor) or Z-VAD (apoptosis inhibitor) pretreatment for 48 h as determined by annexin V/PI staining assay. (B) Effects of different concentrations of GRh3 or pretreatment with a pyroptosis inhibitor or ferroptosis inhibitor before incubation with GRh3-containing medium on cell growth and morphology. Scale bar= 50 μm. (C) IL-1β released into the culture medium was detected by ELISA. (D) IL-18 released into the culture medium was detected by ELISA. (E‒G) Cells were treated with GRh3-containing medium or pretreated with pyroptosis inhibitor, and then, GRh3-containing medium was added and incubated for 48 h. The expression levels of pyroptosis-related proteins were determined by western blot analysis. Data are presented as the mean±SD. ** P<0.01, *** P<0.001 compared with the blank group (0 μM GRh3). ## P<0.01, ### P<0.001 compared with the 40 μM GRh3 group, n=3.

The above experiments showed that a pyroptosis inhibitor (VX-765, Caspase-1 inhibitor) and apoptosis inhibitor (Z-VAD, pancaspase inhibitor) had similar effects on reversing GRh3-induced HT29 and HCT116 cell death ( Figure 2A,B). To determine whether GRh3 induces pyroptosis or apoptosis in colorectal cancer cells, flow cytometry analysis was performed using Annexin V/PI staining. Annexin V/PI staining showed no increase in FITC-labelled apoptosis markers in HT29 and HCT116 cells after GRh3 treatment. Moreover, the nuclear staining marker PI increased as the GRh3 concentration increased, suggesting perforation and other damage to the cell membrane, and this effect could be reversed by pyroptosis inhibitors and apoptosis inhibitors ( Figure 3A). Therefore, GRh3 induces pyroptosis rather than apoptosis in colorectal cancer cells. To more intuitively observe the cell morphology after GRh3 treatment and the reversal effect of the pyroptosis inhibitor and ferroptosis inhibitor, we used an inverted optical microscope to observe the treated cells. We found that as the GRh3 concentration increased, the number of cells decreased, and there were morphological changes, such as cell swelling and cell rupture. Pretreatment with a pyroptosis inhibitor reversed the cell morphological changes induced by GRh3 but did not completely reverse the decrease in the cell number. Moreover, pretreatment with a ferroptosis inhibitor reversed the decrease in the cell number to some extent but did not reverse the morphological changes in the cells ( Figure 3B). These results suggest that GRh3 can induce pyroptosis and ferroptosis in colorectal cancer cells and that these two modes of cell death do not interfere with each other. The ELISA results indicated a dramatic release of IL-1β and IL-18 cytokines into the culture media of the HT29 and HCT116 cells treated with GRh3, and the pyroptosis inhibitor inhibited the release of these cytokines ( Figure 3C,D). Western blot analysis showed that after GRh3 treatment, HO-1 protein expression level was decreased, while NLRP3, Caspase-1 and GSDMD protein expression levels were significantly increased. Meanwhile, the pyroptosis inhibitor (Caspase-1 inhibitor) reversed the increased expression levels of Caspase-1 and GSDMD induced by GRh3 ( Figure 3E‒G). These results suggest that VX-765 attenuates GRh3-induced pyroptosis in HT29 and HCT116 cells. Therefore, GRh3 can induce pyroptosis and inhibit CRC cell proliferation, and this process is positively correlated with GRh3 concentration.

### GRh3 induces ferroptosis in CRC cells *in vitro*

**Figure 4:**
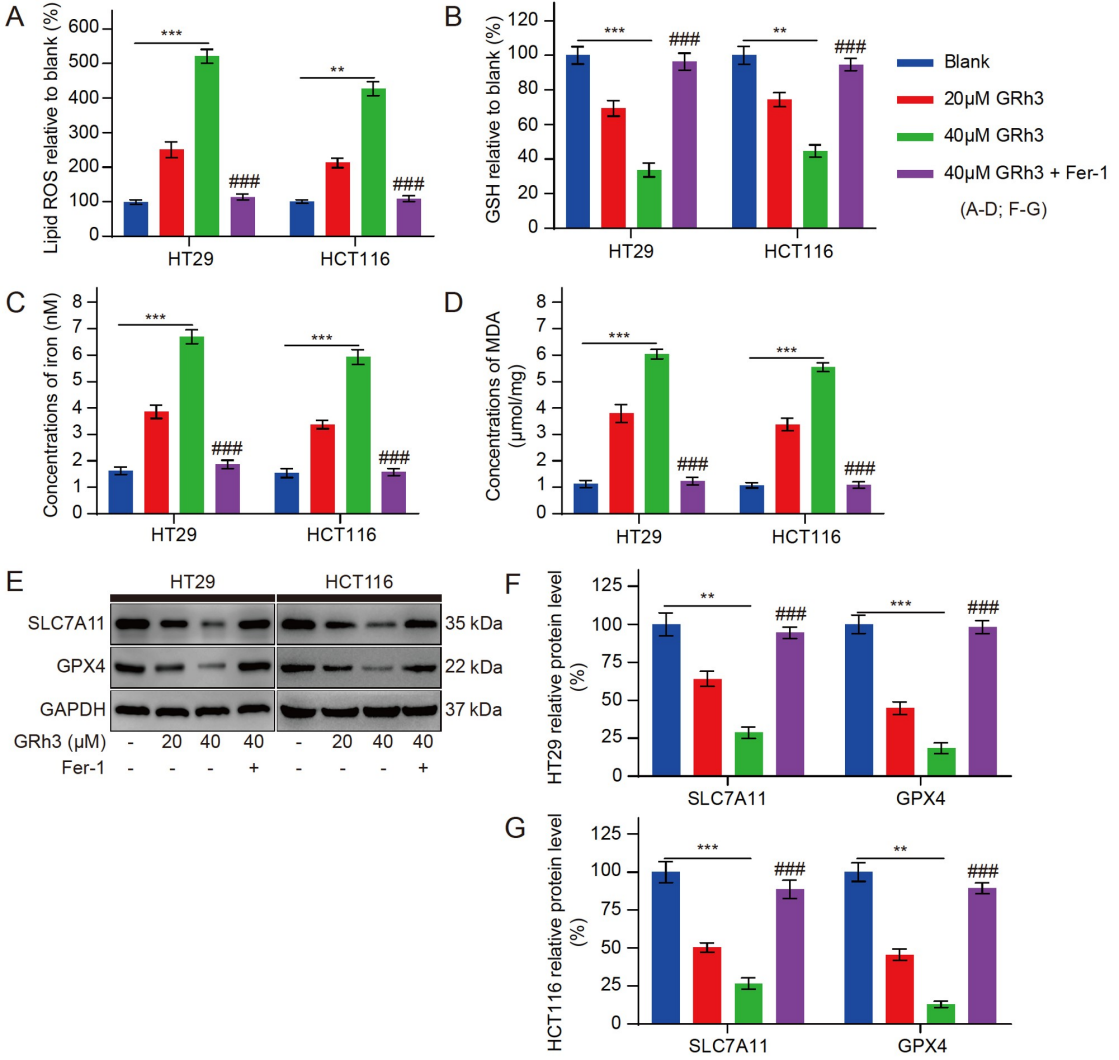
Figure 4 GRh3 induces ferroptosis in HT29 and HCT116 cells (A) Relative concentrations of lipid ROS in HT29 and HCT116 cells exposed to different concentrations of GRh3 in medium with or without Fer-1 pretreatment (4 h) for 48 h. (B) Relative concentrations of GSH in HT29 and HCT116 cells. (C) Iron concentrations in HT29 and HCT116 cells. (D) Concentrations of MDA in HT29 and HCT116 cells. (E) Representative western blots of ferroptosis-related protein expression in HT29 and HCT116 cells. (F,G) Relative expression levels of ferroptosis-related proteins in HT29 and HCT116 cells. Data are presented as the mean±SD. ** P<0.01, *** P<0.001 compared with the blank group (0 μM GRh3); ### P<0.001 compared with the 40 μM GRh3 group, n=3.

To further elucidate the anticancer mechanism of GRh3, we examined the effects of GRh3 on ferroptosis-related indicators. GRh3 significantly induced the cellular accumulation of lipid ROS, iron and MDA, and reduced the concentration of GSH in HT29 and HCT116 cells after 48 h of culture, and these effects were enhanced by increasing concentrations of GRh3 ( Figure 4A‒D). In addition, the increased concentrations of intracellular lipid ROS, iron, and MDA induced by GRh3 were significantly reversed by Fer-1 pretreatment. Furthermore, the changes in GSH concentrations were also reversed. Similarly, to determine whether GRh3 induces ferroptosis, we analyzed the protein expression levels in cultured cells *in vitro*. Western blot analysis showed that as the GRh3 concentration was increased, the expression levels of the ferroptosis-related proteins xCT/SLC7A11 and GPX4 were significantly decreased ( Figure 4E‒G). Furthermore, the expression levels of the GRh3-induced proteins were reversed by Fer-1 pretreatment. These results suggest that Fer-1 attenuates GRh3-induced ferroptosis in HT29 and HCT116 cells. Therefore, GRh3 can induce ferroptosis and inhibit CRC cell proliferation, and this process is positively correlated with GRh3 concentration.

### Pyroptosis and ferroptosis in CRC cells activated by GRh3 are regulated by the Stat3/p53/NRF2 axis

**Figure 5:**
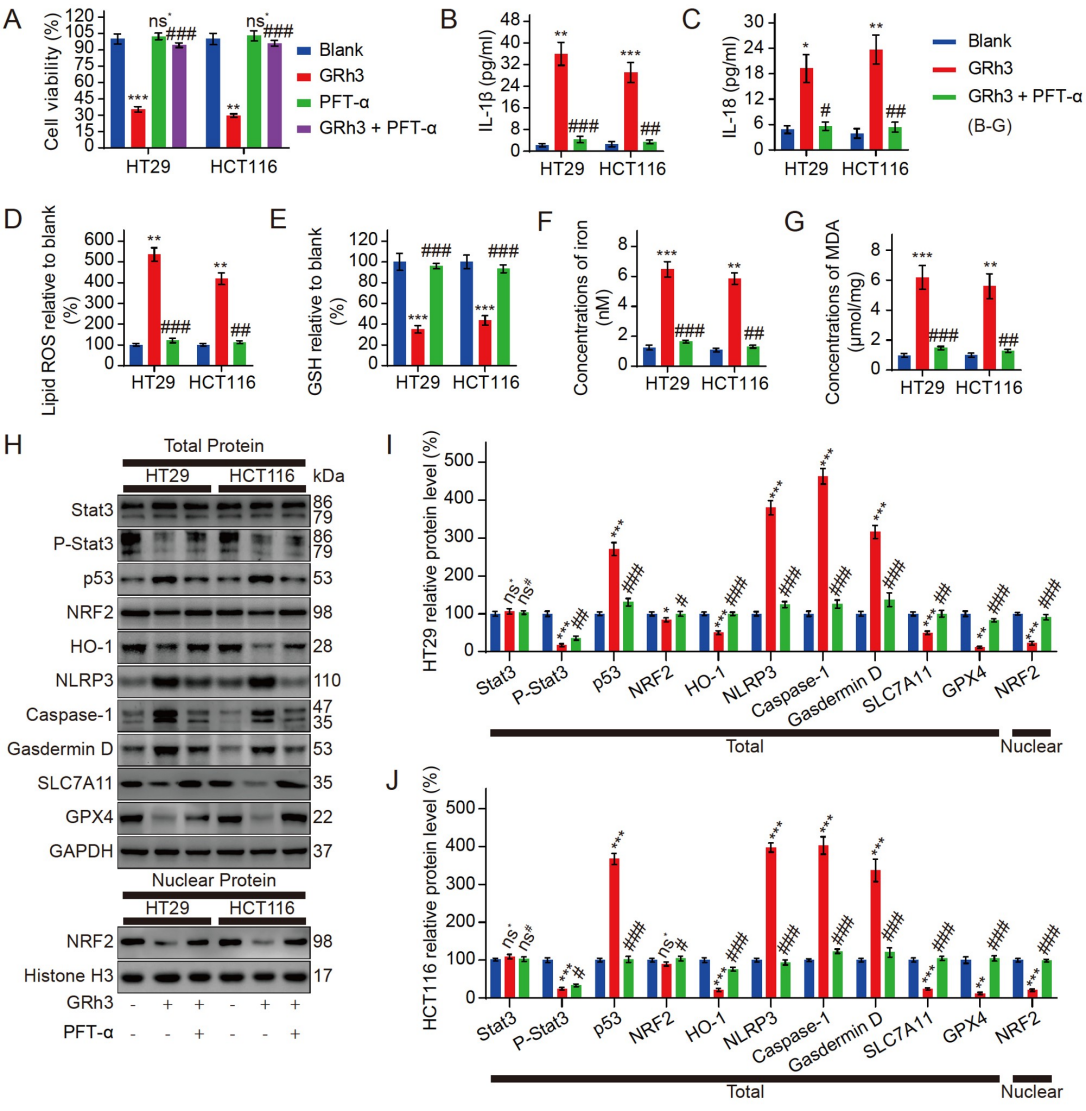
Figure 5 Pyroptosis and ferroptosis in CRC cells activated by GRh3 are regulated by the Stat3/p53/NRF2 axis (A) Viability of HT29 cells and HCT116 cells pretreated with or without PFT-α (p53 inhibitor) followed by treatment with GRh3. (B) IL-1β released into the culture medium was detected by ELISA. (C) IL-18 released into the culture medium was detected by ELISA. (D) Relative concentrations of lipid ROS in HT29 and HCT116 cells. (E) Relative concentrations of GSH in HT29 and HCT116 cells. (F) Iron concentrations in HT29 and HCT116 cells. (G) Concentrations of MDA in HT29 and HCT116 cells. (H) Representative western blots of the Stat3/p53/NRF2 axis, pyroptosis, and ferroptosis-related protein expression in HT29 and HCT116 cells exposed to GRh3-containing medium for 48 h with or without PFT-α pretreatment for 4 h. (I,J) Relative expression levels of Stat3/p53/NRF2 axis-, pyroptosis-, and ferroptosis-related proteins in HT29 and HCT116 cells. Data are presented as the mean±SD. * P<0.05, ** P<0.01, *** P<0.001, vs the blank group (0 μM GRh3). # P<0.01, ## P<0.01, ### P<0.001 vs the 40 μM GRh3 group; n=3.

To investigate whether changes in the Stat3/p53/NFR2 axis are associated with pyroptosis and ferroptosis in CRC cells after GRh3 treatment, we pretreated CRC cells with PFT-α (a p53 inhibitor). The MTT colorimetric assay results showed that PFT-α pretreatment alone had no effect on the proliferative activity of CRC cells, but PFT-α pretreatment reversed the inhibitory effect of GRh3 on CRC cells ( Figure 5A). Meanwhile, after PFT-α pretreatment, the changes in the contents of IL-1β and IL-18 in the medium and the changes in the contents of lipid ROS, GSH, iron and MDA in the cells induced by GRh3 treatment were reversed ( Figure 5B‒G). Western blot analysis showed that the protein expression levels of intracellular p53 and endonuclear NRF2 in the cells pretreated with PFT-α were reversed after GRh3 treatment compared with those in the cells without PFT-α pretreatment, but the changes in Stat3 and p-Stat3 were not significant. Meanwhile, PFT-α pretreatment also reversed the changes in pyroptosis- and ferroptosis-related proteins induced by GRh3 treatment ( Figure 5H‒J). These results suggest that GRh3 induces pyroptosis and ferroptosis in CRC cells via the Stat3/p53/NFR2 axis.

### GRh3 inhibits CRC by activating pyroptosis and ferroptosis *in vivo*

**Figure 6:**
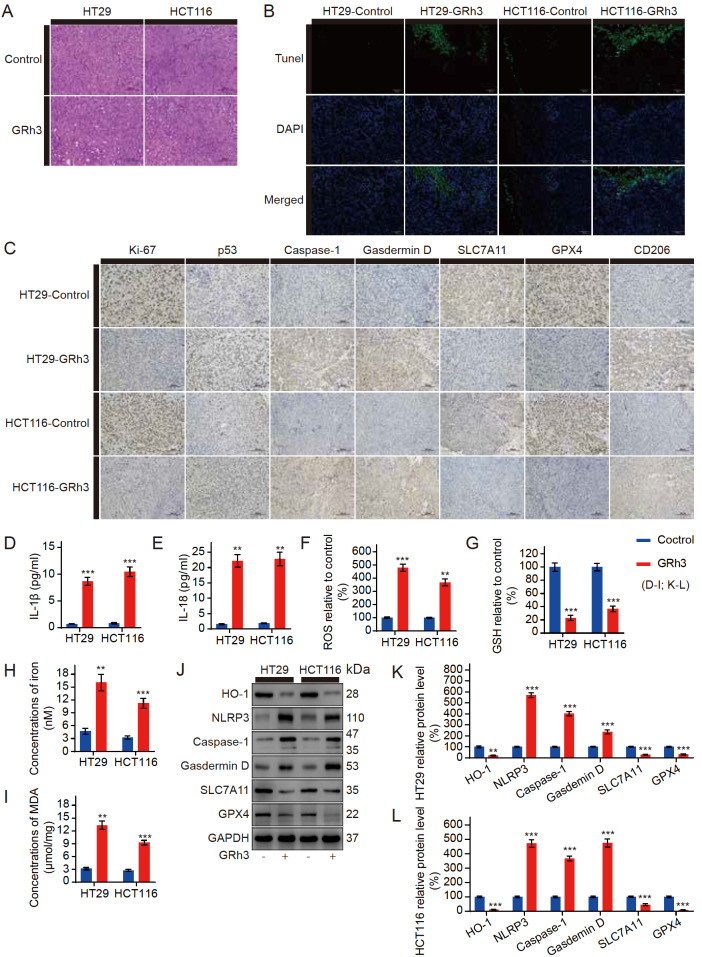
Figure 6 GRh3 induces pyroptosis and ferroptosis in human CRC cells in vivo Mice were treated with or without GRh3. (A) HE staining of tumor specimens. Scale bar= 100 μm. (B) TUNEL staining of tumor specimens. Scale bar= 100 μm. (C) Immunohistochemical staining of tumor specimens. Scale bar= 100 μm. (D) IL-1β concentrations in nude mouse serum. (E) IL-18 concentrations in nude mouse serum. (F) Relative ROS concentrations in tumor tissues. (G) Relative GSH concentrations in tumor tissues. (H) Iron concentrations in tumor tissues. (I) MDA concentrations in tumor tissues. (J) Western blot analysis was performed to analyze the expression levels of pyroptosis- and ferroptosis-related proteins in tumor tissues. (K,L) Relative expression levels of pyroptosis- and ferroptosis-related proteins in HT29 and HCT116 cell xenograft tumor tissues. Data are presented as the mean±SD. ** P<0.01, *** P<0.001, n=3.

To determine whether GRh3 can inhibit CRC growth through ferroptosis *in vivo*, we analysed xenograft tumor tissue samples from mice. Compared with the control, the 20 mg/kg GRh3-treated group was characterized by significant partial necrosis of tumor cells and vacuoles in the tumor tissue ( Figure 6A). Chromatin DNA breaks when cells undergo pyroptosis [20]. Hence, we investigated the effects of GRh3 on cell pyroptosis by TUNEL staining. TUNEL staining revealed that DNA breakage increased after GRh3 treatment ( Figure 6B). The immunohistochemistry analysis of the tumor tissue showed that the mean area that stained positively for Ki-67, which is associated with proliferation, and SLC7A11 and GPX4, which are associated with anti-ferroptosis, were smaller under GRh3 treatment than in the control samples. Meanwhile, the mean area that stained positively for p53, which is associated with inhibiting proliferation, Caspase-1 and Gasdemin D, which are associated with promoting pyroptosis, and CD206, which is associated with inflammatory infiltration, was larger under GRh3 treatment than in the control samples ( Figure 6C). The ELISA results showed that the serum IL-1β and IL-18 concentrations, which are positively correlated with pyroptosis, were increased in the GRh3-treated mice compared with those in the control group ( Figure 6D,E). In the HT29 and HCT116 cell xenograft tumor tissues, the concentrations of ROS, iron and MDA in the GRh3-treated group were higher than those in the control group ( Figure 6F,H,I), while the concentration of GSH was decreased ( Figure 6G), which is consistent with the results of the *in vitro* experiments. To further confirm the increased levels of pyroptosis and ferroptosis in the tumor tissues from the GRh3-treated group, we extracted proteins from the HT29 and HCT116 cell xenograft tumor tissues for analysis. Western blot analysis of the xenograft tumor tissue indicated that the HO-1, SLC7A11, and GPX4 protein expression levels were decreased compared with those in the control, while the NLRP3, Caspase-1, and Gasdemin D protein expression levels were increased ( Figure 6J‒L). These results strongly suggest that both pyroptosis and ferroptosis occur during GRh3-mediated inhibition of tumor growth *in vivo*.

## Discussion

GRh3 is a seminatural triterpene with potent anticancer activity, and its solubility is greater than that of other ginsenosides of the same type; thus, GRh3 has good medicinal potential [14]. Our study showed that GRh3 treatment triggered pyroptotic cell death and ferroptotic cell death in CRC cells via the Stat3/p53/NRF2 axis.

NRF2 has been identified as a significant regulator of the transcription of various antioxidant genes and many other cellular protective genes. The suppression of NRF2-driven heme oxygenase-1 (HO-1) enhances the chemosensitivity of cancer cells [21]. When the intracellular HO-1 expression level is high, pyroptosis-associated NLRP3 expression is inhibited [22] Therefore, the inhibition of HO-1 expression is the key to inducing pyroptotic cell death in tumor cells. NLRP3, as a key factor in pyroptosis, can activate Caspase-1 and then activate the executor of pyroptosis, GSDMD, finally causing pyroptosis [23]. For a long time, the caspase family has been considered a marker associated with apoptosis. Therefore, it cannot be ruled out that over the past few decades, drug-induced pyroptosis has been mistaken for apoptosis. In our experiment, we observed that the pyroptosis inhibitor (VX-765, Caspase-1 inhibitor) and the apoptosis inhibitor (Z-VAD, pancaspase inhibitor) had very similar effects on reversing the inhibitory effect of GRh3 on CRC cells. Therefore, we speculated that Z-VAD only inhibited Caspase-1 but did not affect other sites in the caspase family during the reversal of CRC cell death induced by GRh3. The pyroptosis inhibitor VX-765 partially reversed the reduction in cell number and significantly reversed the change in cell morphology, further confirming that GRh3 inhibited the proliferation of cancer cells by inducing pyroptosis in CRC cells and suggesting that GRh3 also induced CRC cell death in other ways. During the experiment, we found an interesting phenomenon in which HT29 cells grew in a stacked pattern, while HCT116 cells grew in a tiled pattern. Therefore, it was more appropriate to observe the number and morphology of cells by using HCT116 cells. Furthermore, when cells undergo pyroptosis, they release proinflammatory IL-1β and IL-18 into the extracellular space [7]. We also confirmed the presence of pyroptosis in CRC cells treated with GRh3 by ELISA of related cytokines in the culture medium.

It has been proven that the inhibition of NRF2 level could induce ferroptosis in CRC cells to suppress tumor growth [19]. NRF2 regulates xCT/SLC7A11 expression levels and system Xc(‒) activity. A decrease in NRF2 affects the activity of system Xc(‒), depletes GSH, and ultimately leads to decreased GPX4 activity. Decreased GPX4 function affects lipid peroxidation and can induce ferroptosis [19] Lipid ROS, GSH, iron and MDA are important indicators of lipid peroxidation and ferroptosis in cells. When ferroptosis occurs in cells, the intracellular antioxidant index GSH is decreased. However, the two intracellular lipid peroxidation indices, lipid ROS and MDA, and iron increase [10]. In this study, we found that after GRh3 treatment, the changes in these biochemical indices in CRC cells were positively correlated with ferroptosis, and there was a dose gradient effect. These changes were reversed by pretreatment with Fer-1 (a ferroptosis inhibitor). We also observed that Fer-1 pretreatment could reverse the reduction in CRC cells caused by GRh3 to a certain extent but could not reverse the cell swelling and other morphological changes caused by GRh3 treatment. Therefore, we can confirm that in the inhibition of CRC cell proliferation by GRh3, there is not only pyroptotic cell death but also ferroptotic cell death.

The antitumor activities of ginsenoside have been previously linked to the selective inhibition of Stat3 phosphorylation in cancer cells [24]. In cancer cells, p-Stat3 inhibition contributes to the expression of the anticancer factor p53 [25]. The expression of p53 can prevent the nuclear translocation of NRF2 from the cytoplasm to the nucleus and render the antioxidant function of NRF2 ineffective [26]. In this study, we used PFT-α (a p53 inhibitor) to explore the relationship between the Stat3/p53/NRF2 axis and GRh3-induced pyroptosis and ferroptosis in CRC cells. The results showed that the pretreatment effect of the p53 inhibitor was approximately equal to the sum of the pretreatment effects of the pyroptosis inhibitor and ferroptosis inhibitor separately by measuring cell viability. In addition, we found that p53 inhibitor pretreatment significantly reversed the increase in p53 protein expression level and the nuclear translocation of NRF2 in CRC cells induced by GRh3. Furthermore, GRh3-induced pyroptosis and ferroptosis-related protein changes were reversed, but p53 inhibitor pretreatment did not significantly reverse Stat3 phosphorylation. These results suggest that GRh3-induced pyroptosis and ferroptosis in CRC cells are mediated by the Stat3/p53/NRF2 axis. Therefore, pyroptosis and ferroptosis induced by GRh3 are regulated by the Stat3/p53/NRF2 axis.

Based on the *in vitro* experiments, we further verified the antitumor effect and safety of GRh3 *in vivo*. Ki67 is an indicator reflecting tumor proliferation and is closely related to the prognosis of tumor patients [27] After GRh3 treatment, the expression of Ki67 in tumor tissues was significantly decreased. In addition, the HE staining results showed that after GRh3 treatment, the tumor tissue appeared cavitated, indicating necrosis and other changes. Surprisingly, HE staining showed that GRh3 caused no significant damage to the liver and kidney structures, and *in vitro* experiments confirmed that GRh3 had no significant inhibitory effect on normal colorectal cells at the required antitumor concentration (<80 μM). Chromosomal instability and DNA fragmentation occur during pyroptosis [28]. Our results showed that the TUNEL-positive area increased after GRh3 treatment, suggesting that DNA damage was aggravated. Immunohistochemistry, western blot analysis and related biochemical indicator analyses showed that the antitumor mechanism of GRh3 *in vivo* was consistent with that *in vitro*. Pyroptosis is an inflammatory cell death mode closely related to immunity. CD206 is an indicator of tissue immune infiltration [29]. Our results showed that the infiltration area of CD206 in tumor tissues increased after GRh3 treatment, suggesting the activation of pyroptosis. In conclusion, GRh3 can safely exert antitumor effects *in vivo*, and its mechanism of action is consistent with that *in vitro*.

Finally, through a computer simulation of molecular docking, we speculated that GRh3 could effectively and specifically bind to the phosphorylation site of Stat3, preventing the normal phosphorylation process of Stat3, initiating the antitumor process, and ultimately inhibiting the proliferation of cancer cells. Due to the disorder of glucose metabolism in cancer cells, which mainly provide energy to cells in the form of glycolysis [30], these cells are more sensitive to oxidative stress than other normal cells, and the Stat3/p53/NRF2 axis is closely related to the regulation of oxidative stress [ 31, 32] . This may explain why GRh3 has no significant effect on normal cells at antitumor concentrations. Therefore, drugs acting via the Stat3/p53/NRF2 axis are an ideal potential target for antitumor drug development, and GRh3 is a representative of this class of drugs.

**Figure 7:**
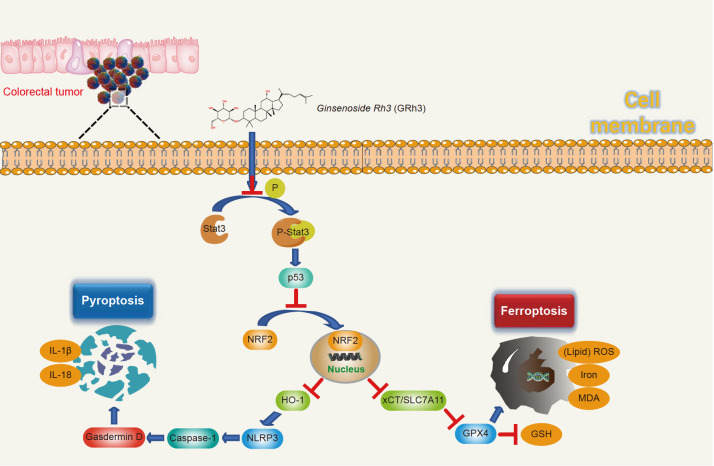
Figure 7 Schematic illustration of the potential underlying mechanism responsible for GRh3-induced pyroptosis and ferroptosis

The mechanism by which GRh3 inhibits proliferation and induces pyroptosis and ferroptosis via the Stat3/p53/NRF2 axis in CRC cells is illustrated in Figure 7. In conclusion, GRh3 inhibits tumor growth via the Stat3/p53/NRF2 axis to induce pyroptosis and ferroptosis in CRC cells. Our study provides a novel paradigm of the antitumor activity of GRh3 in colorectal cancers. We confirmed that GRh3 inhibits tumor growth via the Stat3/p53/NRF2 axis to induce pyroptosis and ferroptosis in CRC cells.
